# Diagnosis of the Lesion in Paraplegia

**Published:** 1875-11

**Authors:** H. C. Wood


### MISCELLANEOUS.

### Diagnosis of the Lesion in Paraplegia.

By H. C. Wood, M. D.

GENTLEMEN:—The patient before us offers himself with the statement that he is paralyzed in the lower limbs, or in other words, is suffering from paraplegia. As paraplegia is not a disease, but a symptom, we must in every case endeavor to determine the cause of the paralysis, or, in other words, the real nature of the disease. Before attempting, this, however, we must be sure that the patient is really suffering from nervous paraplegia, because cases will present themselves in which the supposed palsy is due to general feebleness, to suppression of movements owing to the pain caused by them, to diseases of the muscles themselves, to paralysis of groups of muscles, or to disturbances of sensibility and loss of co-ordinating power. In regard to the diseases of the muscles, it may be stated as an axiom that when the lower limbs are paralyzed without any of the muscles of the trunk or of the upper extremities being affected, the paralysis is not due to disease of the muscles themselves; muscular atrophy, pseudo-hypertrophy, and allied forms of muscular degeneration never, so far as I know, effecting exclusively the lower extremities.

The apparent paralysis in the man before us is not, then, due to muscular degeneration. Neither is it caused by pain, since our patient suffers none whether at rest or in motion; nor are any groups of the leg-muscles especially effected. Is the man then suffering from loss of co-ordinating power, or, in other words, from locomotor ataxia? Watch him as he walks by the aid of a cane. See how his feet glide over the floor. He cannot raise them, but slips them over the smooth surface. The gait is enough of itself to show that he is paraplegic. The man whose muscles retain their vigor, although he cannot control them, lifts his feet hastily and irregularly, but with no feebleness. Laying the man on his back, and testing the muscular power of his legs, we are confirmed in this opinion; the patient is certainly paraplegic.

Having made out the existence of the symptom, before endeavoring to determine its cause let us get out the important facts of the case. These may be summed as follows. First, the attack came on gradually; second, there has been at no time much pain; third, there is decided paresis of the bladder, which followed, not preceded, the paresis of the legs; fourth, the loss of power in the legs is very marked, but not absolute; fifth, the muscles are not very much wasted, and retain, to a fair extent, their electro-contractility; sixth, reflex movements are certainly below rather than above normal, and, so far as can be determined by questioning, never heve been above normal; finally, there is no history of a recent attack of acute disease, and the absence from the gums of the green line of copper-poisoning, and the blue line of lead poisoning as well as the lack of the spongy gums and general tremors and cachexia of mercurial toxæmia, and the general expression of health in the man's countenance, negative the the idea that our patient is at present suffering from a dyscrasia, either toxic or constitutional.

We are now ready to endeavor to discover the cause of the paralysis. To facilitate this, I have written upon the blackboard the three possible sets of causes of paraplegia:

| 1. Organic. | 2. Functional. | 3. Hysterical. |
|---|---|---|
| Diseases of bones. | Anæmic | Hysteria. |
| " membranes. | Reflex. | |
| " cord. | Dyscrasic. | |

The term functional, which is here employed, might be objected to; but do not let us waste time in this: the group is clinically a very useful one, and the name is a matter of little importance. Hysterical paraplegia is undoubtedly functional, but I have separated it because in its diagnostic peculiarities it resembles more closely the organic palsies than those of Group 2.

The important question here naturally arises, are we able to determine from intrinsic characters to which of these groups a given case belongs? Usually, gentlemen, we are, but not always. Let us employ the man before us as an example. In the so-called functional paraplegias the onset is always gradual; in the organic it may be almost instantaneous or very rapid, but probably it is more often gradual. In the hysterical variety the paralysis is usually developed at once. In the present case it is plain that these facts do not aid us in the diagnosis, for the disease developed gradually. The loss of power is still imperfect, and has only arrived at its present degree of completeness by a very slow progress of months. In the organic paraplegias you usually have at some period of the disease indications of a motor or sensory functional excitement in the form of spasm or of pain in the affected limbs. In both the hysterical and the functional varieties you rarely, if ever, have any such indications. There is one especial form of pain, very possibly connected with spasm, which appears to be actually diagnostic of organic diseases of the spinal cord or membranes. It is the sensation of a band or cincture around the waist. Our patient, in answer to the question, states that he has felt at times as though a cord were being drawn so tightly around his body that he must fall in two. Again, in functional paraplegias you do not have distinct disturbance of sensation. Partial or even complete anæsthesia is very common in hysterical paraplegia; but there is one modification of sensibility which appears to rank with the cincture in its diagnostic importance. This is the retardation of the passage of a sensation from the periphery to the centre, so that a very perceptible time elapses between the patient' seeing his feet touch the floor and feeling that they do so. In our case this is not distinctly present, but you see that the man feels upon his legs the compass points as one point, although they are really separated by at least six inches.

Since in this man before us there has been a very distinct cincture present for months, and since there is a distinct loss of sensibility in the legs, and at the same time no general indications, past or present, of a hysterical constitution, it is very positive that there is organic spinal disease. Excitation of the reflex activity is, when present, an almost decisive proof of the existence of organic spinal disease; but in the present instance we can get no aid from this, since the reflex movements are not above par. There is, however, one feature of the case which strongly confirms the opinion already expressed. It is the presence of difficulty in passing urine, evidently due to paresis of the walls of the bladder. Whenever, in the course of a paraplegia, distinct disturbance of the innervation of the bladder occurs, it is very certain that organic disease of the cord or membranes is developing itself.

Our patient is, then, suffering from organic spinal disease. But suppose we had found that the onset had been gradual, that there had been no pain or spasms, that sensibility was not disturbed, that reflex movements were not and had not been increased, that the bladder was unscathed; would it be certain that the patient was not suffering from an organic spinal affection? No, gentlemen; in a case of so long duration as the present the probabilities would be against the presence of a decided lesion of the cord, but not even this could be said if the disease had lasted but a few weeks. In such cases as that which I have imagined, it is necessary to go over the various possible causes of paraplegia *seriatim*. Search if there be a stone in the kidney or bladder; or, in a woman, if there be a uterine tumor to start the chain of phenomena ending in reflex paraplegia. See if there exist any dyscrasia; or, if anæmia be present; look whether the sufferer be hysterical. Very rarely, however, will this diagnosis by exclusion be necessary in practice, except it be in the very earliest stages of the disorder. Attention to the points already detailed will almost always enable the diagnostician to arrive at a rapid and correct conclusion.

Having determined that our patient is suffering from organic spinal disease, it must be our next endeavor to make out the nature of the lesion. The organic causes of paraplegia are diseases of the bones, of the membranes, and of the cord. On examining the spine of the patient we find that it is free from any irregularities and that no evidence of tenderness is elicited by direct pressure or by tapping the patient on the top of the head.

Rosenthal's test of spinal caries consists in passing a pair of electrodes attached to a faradaic battery of some power down the back, one pole being placed each side of the spine. Under these circumstances, if there be any caries or inflamation of the vertebræ, the moment its locality is reached the patient starts or screams from the burning, sticking pain caused by the passage of the galvanic current through the inflamed tissue. I have not found this test as trustworthy as its originator claimed it to be, and as, apparently, it ought to be. I have seen the local pain produced when the cord and not the bony canal was the seat of the disease. In such cases, however, the pain is probably not so severe as where there is commencing or advanced caries of the vertebræ. Moreover, absence of the pain in any case seems to be conclusive evidence of the non-existence of bone-disease. Our patient fails entirely to respond to this test. As, then, there are no discoverable local indications of the presence of diseases of the bony envelopes of the cord, and as there are no constitutional evidences of a scrofulous or syphilitic taint, I think we may narrow the possible seat of the lesion to the cord and its membranes.

The diseases of the membranes and of the cord are as follows:

| MEMBRANES. | CORD. |
| --- | --- |
| Congestion. | Sexual exhaustion. |
| Hæmatorrhachis. | Acute myelitis. |
| Acute meningitis. | Chronic myelitis. |
| Chronic meningitis. | Spinal apoplexy. |
| | Tumors. |

This arrangement of these diseases is a clincally convenient rather than a strictly accurate one. In the so-called congestion of the membranes, the cord is probably also congested very deeply. Further, tumors of the membranes produce the same symptoms as tumors of the cord, and are here included under the general heading of tumors of the cord.

It is plain that in the present instance we may exclude all diseases which are acute in their course or in their onset. This diminishes our possible selection to chronic meningitis, spinal exhaustion, chronic myelitis, and tumors.

That the man is not suffering from a spinal tumor is proven by the condition of the reflex movements. Somewhere at the base of the brain is situated a nerve-centre which controls, or, in other words, inhibits the reflex activity of the spinal cord. Hence, when the vivisector divides the cord of the animal, the reflex movements in the hind legs are greatly increased, and when in man the continuity of the cord is interrupted by the knife of the assassin, or by the growth of a tumor, parallel phenomena result. As reflex movements are not, and, so far as we can learn, never have been, in our patient greater than normal, we dismiss the idea of a tumor. The possible diagnosis has thus been limited to chronic spinal meningitis, sexual exhaustion, and chronic myelitis Is our patient effected with the first of these?

Think a moment of the anatomy of the spinal membranes; excepting the fine, hardly existing pia mater, their relations with the spinal cord are not very intimate,whilst they are brought into the closest union with the spinal nerve-roots which pierce them; consequently, when the membranes are inflamed, the nerve-roots feel much more than the cord the effects of the disorder, and soon come to share in the inflammation; frightful pains and no less frightful spasms, darting and pirouetting through the limbs, mark the excitement not of the membranes, nor of the cord, but of the spinal nerve-roots. No paralysis can appear until sufficient exudation to abolish nerve-function by pressure has taken place. In chronic spinal meningitis very rarely, if ever, is the exudation sufficient to do this, and, consequently, pain and spasm predominate over paralysis even in the advanced stages of the disease. The legs of the patient are not extended and flaccid, but drawn up upon the body, with the muscles hard and tense, and extension impossible, except by application of external force, and then very painful. Plainly, our patient is not suffering from chronic meningitis.

Of chronic myelitis there are two chief varieties—one ending in hardening of the cord, the other in the developments of spots of softened tissue. In the first of these, *sclerosis*, the whole cord or any portion of it may be affected. The process is very similar to that which occurs in cirrhosis, of the liver, consisting essentially of an excessive nutritive activity or chronic inflammation of the connective tissue of the cord, giving rise to a great increase of this tissue, and finally a destruction of nerve-cells and tubules by pressure. This process is essentially a very slow one, and for a long time is associated with a condition of hyperæmia and functional excitement of the spinal centres; hence, frequent local spasms, with or without darting pains, according to the position of the lesion, precede, and often co-exist with, the earlier stages of the paralysis. It is also evident that there must be, during the earlier portions of the disease, an exalted state of reflex activity; moreover, in the majority of cases of spinal sclerosis the membranes share the disorder, and, from the resultant irritation of the nerves, pain and spasm result.

In that form of chronic myelitis which results in softening and breaking down of the nerve-tissues, the membranes are rarely effected, and the cord itself is not during any long period in the condition of chronic hyperæmia and irritation seen in spinal sclerosis. Very early in the disease the nerve cells commence a deterioration which lessons their functional activity. For the reasons just given, the onset of this form of chronic myelitis is a torpid one. The spasms. the pains, the heightened reflex movements of sclerosis, are wanting. Not rarely these two forms of chronic myelitis co-exist, when of course the symptomatology is of a mixed character. If the history given by our patient be correct, it is plain that *any chronic* myelitis which may exist must be chiefly of the softening type.

It also follows from what has been brought forward that the man before us has either chronic myelitis or the affection I have denominated sexual exhaustion. Sexual exhaustion is hardly worthy of being recognized as a distinct disease, for the difference between it and myelitis is probably only one of degree. Still, there seems to me a practical advantage in the term. A man abuses his sexual powers, suffers from paraplegia, comes under teatment and gets well, while a second patient under similar circumstances develops an incurable myelitis. There is no reason for believing that the two cases are really anatomically diverse. There is much reason for clinically separating curable and incurable cases.

Let us pause a moment to see how sexual excesses produce spinal disorder During the sexual act the nerve-centres, and probably especially the lower portions of the cord, are intensely excited. Many of the sexual movements are undoubtedly reflex, and the final orgasm is accompanied by a paroxysm comparable to a spinal epilepsy. It cannot but be that there is an active hyperæmia of the the nerve-centres during the act, and a decided exhaustion of these centres follows as the result of their intense functional activity. Normally, the congestion subsides and the exhaustion is recovered from without any evil results. But if the act be repeated at short intervals, the repeated congestion exhausts the vessels, so that there is developed a condition of chronic congestion along with a constantly increasing exhaustion. The spinal centres are therefore, placed under the most favorable circumstances for the development of a low grade of nutritive changes ending in degeneration and constituting one of the processes of inflammation. If a case is seen very early, before any decided change has occurred, we speak of of it as spinal exhaustion, and the chances of affording relief are good. If, however, the structural degeneration has progressed, then we say the man has chronic myelitic, and the prognosis is very gloomy. Of course there is every shade between the extremes, and it is often impossible to locate exactly a given case.

The patient before us dates his illness to abuse of his generative function; but two years have now elapsed and still the paraplegia slowly deepens. I cannot help believing that he has a chronic myelitis, which will probably resist all treatment. Scattered through his cord are probably spots of destroyed tissue, not sufficient in amount to overwhelm the spinal function entirely, but enough to weaken it greatly. To restore this degenerated tissue, is impossible; it is even very doubtful whether we can arrest the further increase of the lesion.—*Phila. Med. Times.*

